# A clearer view of the insect brain—combining bleaching with standard whole-mount immunocytochemistry allows confocal imaging of pigment-covered brain areas for 3D reconstruction

**DOI:** 10.3389/fnana.2015.00121

**Published:** 2015-09-07

**Authors:** Anna L. Stöckl, Stanley Heinze

**Affiliations:** Department of Biology, Lund UniversityLund, Sweden

**Keywords:** whole-mount labeling, confocal imaging, lamina, pigment, bleaching

## Abstract

In the study of insect neuroanatomy, three-dimensional (3D) reconstructions of neurons and neuropils have become a standard technique. As images have to be obtained from whole-mount brain preparations, pigmentation on the brain surface poses a serious challenge to imaging. In insects, this is a major problematic in the first visual neuropil of the optic lobe, the lamina, which is obstructed by the pigment of the retina as well as by the pigmented fenestration layer. This has prevented inclusion of this major processing center of the insect visual system into most neuroanatomical brain atlases and hinders imaging of neurons within the lamina by confocal microscopy. It has recently been shown that hydrogen peroxide bleaching is compatible with immunohistochemical labeling in insect brains, and we therefore developed a simple technique for removal of pigments on the surface of insect brains by chemical bleaching. We show that our technique enables imaging of the pigment-obstructed regions of insect brains when combined with standard protocols for both anti-synapsin-labeled as well as neurobiotin-injected samples. This method can be combined with different fixation procedures, as well as different fluorophore excitation wavelengths without negative effects on staining quality. It can therefore serve as an effective addition to most standard histology protocols used in insect neuroanatomy.

## Introduction

Three-dimensional (3D) reconstructions of neurons and neuropils have become a standard technique in the study of insect neuroanatomy. Compared to earlier methods that were based on brain sections, reconstructing neuropils in 3D based on whole-mounts has substantially increased the precision of data underlying brain volumetric analyses. Using these techniques, mean neuropil volumes and their variability have been characterized for a range of species (e.g., Rein et al., 2002; Kurylas et al., 2008; El Jundi et al., 2009; Kvello et al., 2009; Mysore et al., 2009; Ott and Rogers, 2010; Heinze et al., 2013), demonstrating, for example, a striking volume difference between the neuropils in the gregarious and solitary locusts (Ott and Rogers, 2010), or revealing differences in brain volume in different casts of ants (Mysore et al., 2009). Moreover, the relative volume of neuropils can also be compared across species to tackle evolutionary questions, such as trade-off between investments in brain areas serving different ecologically relevant functions in different species (O’Donnell et al., 2013; Streinzer et al., 2013; Montgomery and Ott, 2015). Additionally, 3D-reconstructions of brain areas have aided the development of standardized atlases of insect brains, providing common frames of reference for registration of neuronal morphologies (Brandt et al., 2005; Kurylas et al., 2008; El Jundi et al., 2010; Løfaldli et al., 2010; Chiang et al., 2011). In general, 3D-reconstructions of individual neurons have proven to be powerful tools for identifying neuronal projection fields in unstructured brain regions (Heinze et al., 2013; Beetz et al., 2015), for revealing overlap between elements of neuronal networks (Bräunig, 2008; Kvello et al., 2010; el Jundi et al., 2011; Zhao et al., 2014) as well as to compare the detailed anatomy of homologous neurons across species (Heinze and Reppert, 2011). Data on single neurons can be obtained either by intracellularly injecting neurons with dyes or by genetically expressing fluorescent proteins in specific neurons, while neuropil data is predominantly generated by immunocytochemical labeling of synaptic proteins. Both methods result in brain preparations labeled with fluorescent dyes.

To obtain the data required for subsequent 3D-reconstructions, the labeled tissue needs to be accessible for imaging. Routinely, confocal microscopy is used to acquire stacks of optical sections that are then used as the basis for reconstructions. As images have to be obtained from whole-mount, i.e., unsectioned brain preparations, pigmentation on the brain surface poses a serious challenge to imaging. In insects, this is a major problem for the first visual neuropil of the optic lobe, the lamina. This neuropil is obstructed by pigments of the retina as well as by the pigmented fenestration layer. In some species, the stemmata, remnants of larval eyes (Paulus and Schmidt, 1978) that are visible as pigmented spots on the posterior surface of the brain, are another obstacle for imaging. To date, the most common approach to image pigment-obstructed brain regions has been to physically remove the pigment, carefully trying to not destroy the underlying tissue, which in most species is a challenging task. In the case of the lamina, this has led to either omitting this neuropil from volumetric studies (*Drosophila*, Rein et al., 2002; Ito et al., 2014; honeybee, Brandt et al., 2005; Rybak et al., 2010; *Heliothis* moth, Kvello et al., 2009; red flower beetle, Dreyer et al., 2010; desert locust, Kurylas et al., 2008), or to including it at much lower sample size compared to other neuropils (hawkmoth *Manduca sexta*, El Jundi et al., 2009). Often, problems with obtaining a completely unobstructed lamina become apparent in figures (e.g., Montgomery and Ott, 2015). Consequently, the first processing stage for visual information is not routinely included in brain atlases, despite its crucial role in shaping neural responses to visual stimuli, such as spatial filtering of visual information (Dubs et al., 1981), and relaying of signals into parallel visual pathways (Strausfeld and Lee, 1991; Meinertzhagen and Sorra, 2001; Joesch et al., 2010; Schnaitmann et al., 2013).

Both problems described, i.e., obstructed lamina and pigmented stemmata, occur simultaneously in the brains of lepidopteran insects. In the work presented we have thus used a species of hawkmoth (*Deilephila elpenor*) to develop a technique aimed at the removal of this pigmentation. In vertebrates, hydrogen peroxide bleaching is routinely used for microscopic imaging of whole-mount retinae (Ullmann et al., 2012), while a recent study has provided the proof-of principle that bleaching in insect brains is compatible with immunohistochemical techniques (Smolla et al., 2014). We therefore integrated an optimized bleaching step into standard immunohistochemical protocols used for quantitative insect neuroanatomy. In short, by bleaching the brains with hydrogen peroxide, we successfully removed the detrimental effects of the pigmented tissue, both for imaging neuropil as well as for imaging dye injected single neurons in the moth lamina. This treatment did not have any negative influence on the synapsin or neuron staining quality. This work should therefore open up the possibility to include this major processing center for visual information in brain atlases alongside all other neuropils.

## Materials and Methods

### Animals

In this study, a species of hawkmoth (Lepidoptera: *Sphingidae*) was investigated: the nocturnal elephant hawkmoth *D. elpenor.* Pupae of this species were purchased from Neil West, a collector in Newark, UK and kept at 5° to simulate winter for up to eight months. They were stimulated to eclose by transferring them to room temperature. Moths were fed with 10% sugar solution and kept in flight cages in a 14:10 day:night light regime for 2–14 days before being used for experiments.

### Histology

#### Anti-Synapsin Labeling

**Figure 1:**
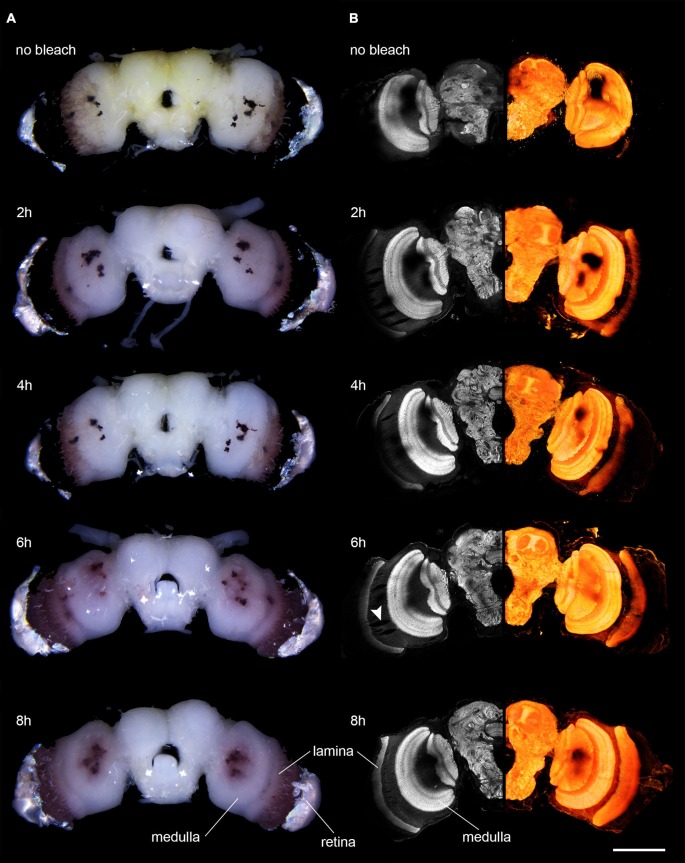
**Microscopic images of anti-synapsin labeled brains of *D. elpenor* bleached with hydrogen peroxide for different lengths of time (duration indicated above each image). (A)** Brains under bright-field illumination. Note the decreasing density of pigment in the vicinity of the retina. **(B)** Confocal image data of the brains shown in **(A)**. Left column: single optical sections of anti-synapsin labeled brains (10x objective). Right column: volume rendering of complete image stacks of the same brain as in the corresponding image in the left column, revealing the 3D structure of the neuropils. Note the decreasing presence of shadowing effects from pigments with increasing bleaching duration. The arrowhead points to artifacts potentially induced by the peroxide treatment. Scale bar: 500 μm.

Whole-mount staining protocols using a monoclonal anti-Synapsin antibody (obtained from Dr. W. Buchner, Cat# SYNORF1 (*Drosophila* synapsin I isoform), RRID: AB_2315426; Klagges et al., 1996) were adapted from staining protocols described in Heinze and Reppert (2012) and Ott (2008). Brains were dissected in chilled HEPES-buffered saline (HBS: 150 mM, NaCl; 5 mM KCl; 5 mM CaCl2; 25 mM sucrose; 10 mM HEPES (N-[2-ydroxyethyl]piperazine-N0-[2-ethanesulphonicacid]; pH 7.2); Ott, 2008), trachea on the brain surface and peripheral layers of the retina were carefully removed, leaving the pigmented fenestration layer intact (Figure 1A). Brains were fixed overnight at room temperature in Zinc-formaldehyde fixative (0.25% [18.4 mM] ZnCl_2_, 135 mM NaCl, 35 mM sucrose, 1% paraformaldehyde (PFA); Ott, 2008). Subsequently, the brains were rinsed 8 × 20 min in HBS and bleached by incubating in a fresh solution of 10% hydrogen peroxide in 0.05 M Tris-buffered saline (Tris-HCl) for either 2, 4, 6, or 8 h. Brains that were not bleached were kept in Tris-HCl for 4 h. After the brains were washed 3 × 10 min in Tris-HCl they were treated with a fresh mixture (20:80) of dimethyl sulfoxide (DMSO)/methanol for 85 min to increase tissue permeability. After washing 3 × 10 min in Tris-HCl, preincubation was performed with 5% normal goat serum in 0.01M phosphate buffered saline (PBS) with 0.3% TritonX-100 (PBT) over night at 4°C and incubated with 1:25 anti synapsin antibodies (in PBT with 1% NGS) for 5 days at 4°C. After washing for 8 × 30 min in PBT, the secondary antibody, goat anti-mouse conjugated to Cy5 (Cy5-GAM, 1:300; Jackson ImmunoResearch, West Grove, PA, USA; catalog number 115-175-146) was applied for 5 days at 4°C. Successively, brains were washed 6 × 30 min in PBT and 2 × 30 min in PBS, dehydrated in an ethanol series of increasing concentrations (50, 70, 90, 95 and 100%; 15 min each). Brains were cleared in methyl salicylate, initially applied as a fresh mixture (1:1) of ethanol and methyl salicylate for 15 min (while ethanol was allowed to evaporate), and then as pure methyl salicylate for an additional minimum time of 45 min. Brains were mounted between two coverslips using Permount (Electron Microscopy Science, Hartfield, PA, USA). Plastic spacers (Zweckform No.3510, Germany) were used to prevent squeezing of the brains.

#### Neurobiotin Injections

For neurobiotin labeling, moths were restrained by taping the thorax tightly to a holder to prevent movement of the flight muscles. The head and thorax were fixed with wax, and the head capsule was opened to expose the brain. Neurobiotin (Vector Laboratories, Burlingame, UK) crystals were applied to the tip of a borosilicate micropipette, which was inserted manually into the target brain region. The brain was dissected and fixed overnight at 4°C in a fixative containing 4% PFA, 0.25% glutaraldehyde, and 2% saturated picric acid (in 0.1 M phosphate buffer). Brains were washed 4 × 15 min in 0.1M PBS, bleached in 10% hydrogen peroxide for either 30 min, 1 and 2 h, washed in Tris-HCl 3 × 10 min, and then incubated with Cy3-conjugated streptavidin (1:1000; Jackson ImmunoResearch, West Grove, PA, USA; catalog number 016-160-084) for three days at 4°C. After incubation, brains were rinsed 6 × 15 min in PBT and 2 × 20 min in PBS, dehydrated in an ascending ethanol series (50, 70, 90, 95 and 100%; 15 min each), treated with a 1:1 mix of 100% ethanol and methyl salycilate for 15 min, and eventually cleared for at least 45 min in pure methyl salycitate. As for antibody labeled preparations, brains were mounted in Permount between two coverslips, using plastic spacers to prevent squeezing of the brains.

### Imaging

Photographs of bleached and non-bleached brains were taken in phosphate buffer using the 2× objective (SHR Plan Apo; Nikon) on a SMZ25 Nikon stereo-microscope with a Nikon DS-Ri1 camera.

Anti-synapsin-labeled whole-mount preparations were imaged using a 633 nm HeNe laser on a confocal microscope (LSM 510 Meta, Zeiss, Jena, Germany) using a 10× objective (Plan Neofluar 0.45 water immersion; Zeiss). We scanned at a frame size of 1024 × 1024 voxels with optical sections every 3 μm. The resulting voxel size was 1.24 × 1.24 × 3 μm with a field of view covering 1269.8 × 1269.8 μm. Detector range was set to 646 nm 753 nm, pinhole to 1 airy unit, and pixel dwell time was 1.6 μs. Detector gain and laser power was adjusted for each preparation to obtain optimally exposed images (gain range: 450–550; laser range: 3.5–8%). The refractive index mismatch between immersion medium (immersion oil with RI 1.34) and the mounting medium (RI 1.52) was corrected by rescaling the images in the *z*-dimension by a factor of 1.134 before further analysis.

Neurobiotin-injected brains were imaged with the 561 nm DPSS laser, using either a 10× objective (Plan Neofluar 0.45 water immersion; Zeiss) or a 25× objective (LD LCI Plan-Apochromat 25×/0.8 Imm Corr DIC; Zeiss). For imaging with the 10× objective, settings were identical to those used for antibody labeled brains. The only difference was the detector range, which was set by a lowpass filter allowing all wavelengths above 575 nm to pass. For scans using the 25× objective, we scanned at a frame size of 1024 × 1024 voxels with optical sections every 1 μm. The resulting voxel size was 0.50 × 0.50 × 1 μm with a field of view covering 512 × 512 μm. The detector range was set by a lowpass filter (575 nm), pinhole was set to one airy unit, and pixel dwell time was 3.2 μs. Detector gain and laser power was adjusted for each preparation to obtain optimally exposed images (gain range: 450–500; laser range: 2.5–6%).

For all immunolabeled preparations, several image stacks had to be obtained to cover the entire brain. These image stacks were aligned to a common coordinate frame using the software Amira 5.5.3 (FEI, Hillsboro, Oregon, USA). For visualizing the 3D outline of the imaged brains, volume rendering was performed using the “volren” tool in Amira. For neurobiotin-injected brains, maximal-intensity projections of representative sections of the obtained image stacks were carried out.

## Results

**Figure 2:**
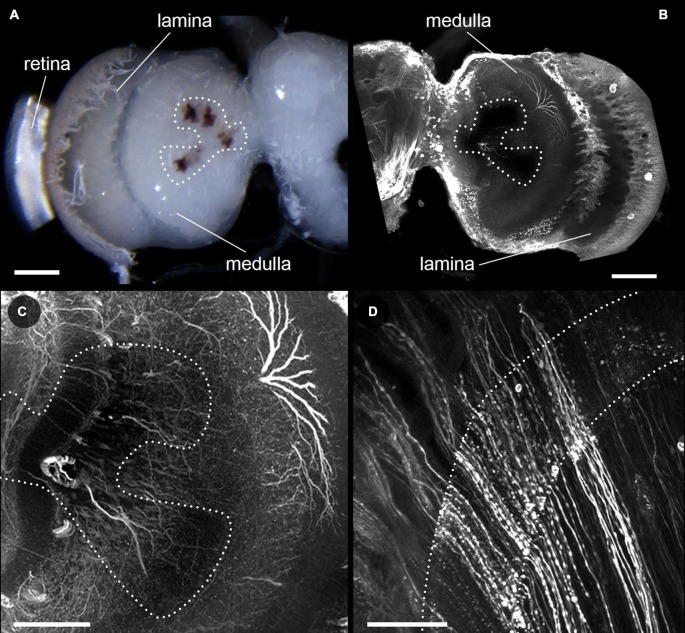
**Effects of bleaching on neurobiotin injected brains. (A)** Light microscopic image of a neurobiotin injected brain of *D. elpenor* bleached with hydrogen peroxide for 2 h. **(B–D)** Single optical sections from a confocal image stack, showing neurobiotin injected neurons in the optic lobe imaged with a 10x objective **(B)**, as well as with a 25× objective in the lobula plate and medulla **(C)**, and the lamina (dotted outlines, **(D)**). **(B)** and **(C)** are taken from the brain shown in **(A)**. Note that neurites located underneath the stemmata (dotted region in **(A–C)**) are well-visible when imaged with the high magnification objective. Scale bars: 200 μm **(A,B)**, 100 μm **(C)**, 50 μm **(D)**.

Bleaching of the brains of the hawkmoth *D. elpenor* led to a reduction of visible surface pigment in conventional bright-field light microscopy. After bleaching for 2 h, the amount of visible pigment within the neural sheath covering the optic lobes (especially surrounding the medulla and lamina) and the central brain was markedly reduced (Figure 1A). In brains prepared for anti-synapsin-labeling, the pigment of the stemmata, as well as the pigment of the fenestration layer and retina became notably lighter (from almost black to reddish-brown) after prolonged bleaching times of 6–8 h (Figure 1A). Longer bleaching times (16 h) lead to no further reduction in the pigment intensity when observed with conventional microscopy. Surprisingly, when brains were fixed for neurobiotin labeling, the visible effects of bleaching occurred much faster (no further visible changes after 2 h in four out of four preparations) and was almost complete (Figure 2A). The effects of bleaching therefore are strongly dependent on the type of fixation.

We next examined whether the observed visual effects would result in improved imaging capabilities using confocal microscopy. When we scanned a non-bleached anti-synapsin-labeled preparation, the lasers penetrated neither the retinal pigment nor the pigment of the fenestration layer. Therefore, the obtained image brightness in the underlying lamina was zero (Figure 1B). With successively longer duration of bleaching, the lamina became increasingly visible, in line with the visual disappearance of the dark pigment. After 6 h of bleaching the complete lamina was visible. However, the signal was still slightly dimmed by the overlying pigment. This issue was resolved after 8 h of bleaching, when the lamina was fully visible. A similar picture arose for the pigment of the stemmata. They most strongly blocked the view of the underlying tissue when the brains were not bleached, but transmission improved with increased bleaching time. Even though imaging of the underlying neuropil was possible in bleached brains, the stemmata never became fully transparent to the lasers.

Additionally to the intended effects on the pigmented surfaces of the brain, bleaching also substantially reduced the unspecific background staining in anti-synapsin-labeled brains, in particular in the periphery of the brain. This resulted in clearer images of the stained neuropils (Figure 1B, compare first and second row). In general, bleaching did not have any negative effect on the quality of the staining, independently of the incubation times used (Figure 1B). In three out of nine bleached brains however, we found small effects on tissue integrity that we did not observe in non-bleached brains. In parts of these samples the tissue was locally ruptured by what looked like enclosed air-bubbles (Figure 1B, arrowhead). These mainly formed within the first optic chiasm between the lamina and medulla and only in very rare cases extended into the neuropils themselves. In general, the overall bleaching time did not correlate with this effect.

After analyzing the effects of bleaching on antibody-labeled brains prepared with Zinc-formaldehyde fixative and imaged at long wavelengths (excitation at 633 nm), we examined whether the positive effects of increased laser penetrability would also hold up for a different fixation protocol and shorter excitation wavelengths (561 nm). In line with our initial observations using bright-field microscopy, bleaching of brains also worked well in combination with a standard neurobiotin-injection protocol aimed at imaging of individual neurons. The neurobiotin-labeled neurons were clearly visible in all bleached preparations, and no effects other than the reduction of pigment density were observed (Figure 2B). Neurons in the lamina, which are completely obstructed from the lasers by pigment in untreated brains, became clearly visible in the preparations bleached for 2 h (Figure 2D). The same effect was also observed for neurons directly underlying the stemmata in the lobula and lobula plate of the optic lobe (Figure 2C). Bleaching therefore improves the penetrability of retinal and stemmata pigment for excitation and emission light in confocal imaging across a broad range of wavelengths (at least from 561 nm to 753 nm). Morevoer, it is effective in combination with two substantially different fixatives (PFA/glutaraldehyde/picric acid vs. Zinc-Chloride/PFA).

## Discussion

Following up on previous work by Smolla et al. (2014), we have developed a technique for the removal of pigments on the surface of insect brains by chemical bleaching. These pigments normally block the light-path for excitation and emission light during confocal fluorescent microscopy and have prevented the inclusion of the first optic neuropil, the lamina, in most insect brain atlases (*Drosophila*, Rein et al., 2002; Ito et al., 2014; honeybee, Brandt et al., 2005; Rybak et al., 2010; *Heliothis* moth, Kvello et al., 2009; red flower beetle, Dreyer et al., 2010; desert locust, Kurylas et al., 2008), as well as obstructing imaging of individual neurons in the lamina. Our technique proved to be suitable for enabling imaging of pigment-obstructed regions of insect brains using both anti-synapsin-labeled as well as neurobiotin-injected samples. We showed that, in principle, this technique can be combined with different fixation protocols, as well as different fluorophore excitation wavelengths and should therefore be applicable to most standard histology protocols used in insect neuroanatomy.

Surprisingly, we observed different efficiencies of hydrogen peroxide bleaching with different fixative solutions. While pigments were completely bleached within 2 h using a purely cross-linking fixative (PFA/glutaraldehyde/picric acid), it took up to 8 h using Zinc-Chloride containing fixative. One reason for this discrepancy might be that Zinc-Chloride precipitates the cytoplasm (Ott, 2008). While increasing the penetrability of the tissue for antibodies, this effect might create dense, cytoplasmic aggregates of the pigment, which are likely less accessible for the bleaching agent.

To a smaller extent, we also observed variability of bleaching efficiency between individual brain preparations stained with the same protocol. These effects were similar in magnitude to the normally observed variability of immunolabeling and likely occur for similar reasons, e.g., variation in the strength of tissue fixation due to slight variations in fixation duration, dissection times, and age of animals. Overall, by adjusting the duration of hydrogen peroxide exposure, effective bleaching was ultimately obtained with both protocols used. This suggests that the method is applicable to a wide range of fixation techniques and can be extended to insect species with different brain volumes and different pigment densities compared to the hawkmoth species examined. Moreover, since the eyes of *D. elpenor* have been reported to contain the two most common pigment classes in insects, ommin and xanthommatin (Butenandt et al., 1960; Linzen, 1974), this protocol should be effective in the majority of insect species, who share either one or both pigments in their eyes (Linzen, 1974).

Notably, we did not observe any negative effects of bleaching on overall staining quality. To the contrary, in our preparations the signal-to-noise ratio of anti-synapsin labeling improved with bleaching due to reduced background fluorescence in the periphery of the brains (Figure 1B, top panel compared to second row). This effect might be explained by the finding that hydrogen peroxide blocks the effects of endogenous peroxidases (Li et al., 1987), which can remain active in preparations with mild fixation. It is also possible that the hydrogen peroxide helps reduce double bonds in the tissue, similar to sodium borohydride, which results in reduced autofluorescence (Seguela et al., 1984). As background fluorescence varies strongly with staining protocol and species (Buchwalow and Böcker, 2010), it remains to be shown whether this effect is consistent across insects and staining methods. As the only negative effect of our treatment, we observed minor, spherically expanded ruptures in the tissue within the first optic chiasm in some bleached brains (e.g., Figure 1B, arrowhead). We hypothesize that mechanical ruptures in the tissue during dissection are exaggerated by penetrating gas produced as a result of the bleaching reaction. The parallel organization of axons within the optic chiasm might be particularly susceptible for lateral shearing during dissection and therefore feature this effect most prominently. The severity of these effects was not correlated with the duration of bleaching, and they can be likely kept to a minimum by careful dissection procedures. Bleaching duration can thus be freely adjusted to the requirements of various protocols and species without obvious risks of adverse effects on staining quality and tissue preservation.

## Concluding Remarks

Our technique provides a simple method to access pigment-obstructed areas of insects brains through chemical bleaching with hydrogen peroxide. The lamina of the optic lobe in particular is now routinely observable with confocal microscopy, as difficult mechanical removal of pigments is no longer required. Our method can be combined with different fixation methods, as well as different labeling approaches and laser wavelengths. Even though bleaching times will have to be adjusted for different species and staining techniques, our approach appears to be widely applicable for insect neuroanatomy. Since we did not observe any negative effects on staining quality correlated with increased bleaching time, the observed reduction of unspecific background staining might even warrant the introduction of a bleaching step into standard histology protocols.

## Author Contributions

AS and SH designed the study, analysed and interpreted the data. AS acquired the data. AS and SH drafted and revised the manuscript critically for important intellectual content, both approved the final version to be published, and are accountable for all aspects of the work.

## Funding

This study was funded by a Junior Researcher Grant to SH by the Swedish Research Council (award number: VR 621-2012-2213) as well as by grants from the Swedish Research Council (award number: VR 621-2012-2205), and the Air Force Office of Scientific Research (AFOSR, award number FA9550-12-1-0237) supporting AS. The funding sources were neither involved in the work on this study nor in the writing of this article.

## Conflict of Interest Statement

The authors declare that the research was conducted in the absence of any commercial or financial relationships that could be construed as a potential conflict of interest.
